# Supplementary material for: An analysis of obstetric practices and outcomes in a deep rural district hospital in South Africa
Source: PLoS One. 2022 Jan 4;17(1):e0262269. doi: 10.1371/journal.pone.0262269 (PMC8726481; doi:10.1371/journal.pone.0262269)
Supplement: S1 File — (PDF) [file pone.0262269.s001.pdf]

Gmail

Adam Asghar <adam.asghar@gmail.com>

Perinatal M&M

1 message

Kelly Gate <kellygate@gmail.com> 3 February 2020 at 15:07
To: Adam Asghar <adam.asghar@gmail.com>

Perinatal M&M since 2012.xlsx
128K
