# Supplementary material for: An analysis of obstetric practices and outcomes in a deep rural district hospital in South Africa
Source: PLoS One. 2022 Jan 4;17(1):e0262269. doi: 10.1371/journal.pone.0262269 (PMC8726481; doi:10.1371/journal.pone.0262269)
Supplement: S2 File — (PDF) [file pone.0262269.s002.pdf]

THE OBSTETRIC INTRA-PARTUM PRACTICES AND THE MATERNAL/PERINATAL OUTCOMES AT A DEEP RURAL DISTRICT-LEVEL HOSPITAL IN NORTHERN KWAZULU-NATAL, SOUTH AFRICA

**STUDENT NAME:**	Adam Asghar
**STUDENT NUMBER:**	219094030
**COURSE NAME:**	Master of Medicine
**DISCIPLINE:**	Family Medicine
**COURSE CODE:**	MMD-FM
**SUPERVISOR:**	Dr TC Nkabinde
	Prof M Naidoo
**DATE OF SUBMISSION:**	24 JULY 2019

CONTENTS

INTRODUCTION **3**

Research Question 4

AIM AND OBJECTIVES **5**

Overall Aim 5

Specific Objectives 5

LITERATURE REVIEW **6**

RESEARCH DESIGN AND METHODS **9**

ETHICAL CONSIDERATIONS **12**

WORK PLAN **14**

ACKNOWLEDGEMENTS **15**

REFERENCES **16**

APPENDICES **18**

Appendix 1: Data Collection Tool 18

Appendix 2: Gantt Chart 19

Appendix 3: Provincial Referral Criteria 20

Appendix 4: Robson Group and Lucas Class 23

Appendix 5: Bethesda Hospital Caesarean section audit tool 24

Appendix 6: Draft letter to Medical Manager of Bethesda Hospital 25

INTRODUCTION

Selection of the mode of obstetric delivery can be a significant determinant of the maternal and perinatal outcomes of a pregnancy, irrespective of the antenatal course.[1] The selection features of mother-foetus pairs who would clearly benefit from a particular mode of delivery are well-established, with some regional variations in practice.[2] However, application of these practices is not consistent, especially in resource-constrained environments, with consequent failure to improve outcomes.[3, 4]

It is known that caesarean delivery (CD) is a potentially life-saving obstetric intervention, but one which has many potential risks, as compared to vaginal delivery. Internationally, there has been a focus on ensuring that CDs are performed only when indicated, and on increasing access to CD for populations that currently have limited access[5].

Target CD rates (CDRs) are contentious, but, the right CDR is the one that results in the lowest maternal and perinatal morbidity and mortality, in the context of respectful obstetric care. In South Africa, a variety of health system initiatives (Essential Steps in the Management of Obstetric Emergencies, Minimum Standards for Safe Caesarean Delivery, Helping Babies Breathe) have been implemented to minimise peri- and post-delivery risks to the patients being cared for, irrespective of the site at which the care is provided.[6]

The latest reports released by the National Committee for Confidential Enquiries into Maternal Deaths ("Saving Mothers"), and the National Perinatal Morbidity And Mortality Committee ("Saving Babies"), show that maternal and perinatal mortality remain unacceptably high. Caesarean delivery contributes to maternal deaths through haemorrhage, anaesthesia, CD-associated sepsis and thromboembolism in the index pregnancy, and makes for higher-risk deliveries in the future. Conversely, failure to offer CD timeously, contributes to maternal deaths through pregnancy-related sepsis, haemorrhage and hypertension, and perinatal deaths through birth asphyxia, antepartum haemorrhage, hypertension and birth trauma.[7, 8]

Globally, the proportion of births occurring by CD as opposed to vaginal delivery is on the rise.[5, 9] This trend is mirrored in South Africa (with the national CDR increasing from 12.7% in 2001/2 to 26.2% in 2015/16), but has been accompanied by an increase in maternal mortality specifically related to bleeding during or after CD. The case-fatality rate (CFR) for bleeding during or after CD was ~25 in 2008 and has risen to ~30 in 2016; thus there is increased focus at policy level on making CD a safe intrapartum practice.[7, 10, 11]

The Saving Mothers reports note that maternal mortality related to CD is highest in rural provinces, and that the CFR for CD relative to that for vaginal delivery is 1.4 times higher at primary healthcare level than the national relative CFRs for mode of delivery.

It is vital to note that rural environments have multiple challenges. Robust quantitative data based on findings of the 2009 and 2010 South African General Household Surveys exist to detail the

challenges South African rural patients experience in accessing healthcare in general, which include availability (85% urban vs 60% rural), affordability and acceptability (the latter positively associated with rurality, likely due to perception bias created by lack of choice).[12] Moving beyond access to the nearest health facility, it is noted that South Africa's rural areas are home to 43.6% of the country's population, but are only served by 12% of the country's doctors and 19% of its nurses.[13] Looking at the effect of rurality on accessing specialist care at a higher level, Kong[14] found a significantly more complicated clinical course for patients with acute appendicitis in KwaZulu-Natal, if they originated from a rural primary care facility, compared to patients originating from an urban primary care facility.

Although the reports from the ministerial committees are derived from in-depth analysis of deaths throughout the country, they provide generalised conclusions. What has not been done recently is to analyse a variety of outcomes specifically at rural primary healthcare level, where around a third of South African mother-foetus pairs will have their delivery managed (this proportion is based on the catchment population of district hospitals serving rural and urban populations).[15] The gap in this knowledge will be addressed by this research.

Since 2015, the clinical team at Bethesda Hospital has audited CDs, explored the phenomena of decision-making and timing of CDs, and through this exercise, identified areas for improvement.

It is not clear whether this auditing practice has made a positive impact on maternal and perinatal outcomes; Allanson et al.[16] noted that quality-of-care audits do not necessarily improve outcomes. However, one conclusion from the practice is the importance of auditing vaginal deliveries to identify missed opportunities for a CD or operative vaginal delivery.

The primary aim of this study is to perform an analysis of both vaginal deliveries and CDs conducted at Bethesda Hospital, and thus compare maternal and neonatal outcomes with obstetric practices at this rural district-level hospital. This is on the background of maternal and perinatal care being priorities for the National Department of Health.

Research Question

What are the obstetric intra-partum practices and the maternal/perinatal outcomes at a deep rural district-level hospital in northern KwaZulu-Natal, South Africa?

AIM AND OBJECTIVES

Overall Aim

The aim of this study is to describe and compare the maternal and perinatal outcomes related to the obstetric intra-partum practices, at Bethesda Hospital in 2018.

Specific Objectives

1. To describe the demographic and clinical profile of the study population;
2. To describe intra-partum practices at Bethesda Hospital in 2018;
3. To compare the maternal and perinatal outcomes at Bethesda Hospital in 2018 against the modes of delivery.

LITERATURE REVIEW

Introduction

The literature review was conducted using PubMed and Google Scholar, entering the following Medical Subject Headings: “Delivery, Obstetric”, “Cesarean Section”, “Maternal Mortality”, “Perinatal Mortality” and “Hospitals, Rural”. Publications retrieved were screened for relevance according to the title and/or abstract, and if deemed relevant, were then scrutinised. References of scrutinised articles were also reviewed for relevance.

Obstetric practices in a rural low-resource setting

Although not specifically analysing South African trends, it has been found that CDRs are uniformly lower across the world in populations within individual countries who are of a lower socioeconomic status, and residing in a rural area.[17] Caesarean delivery rate can be used as a marker of a population’s access to CD as an obstetric intervention, which thus suggests that rural patients have poorer access to potentially life-saving obstetric surgery on a global level.[18] In South Africa, the disparity of CDR between the richest and poorest quintiles of the population was 3.4-fold in 1998 (this is comparable to Namibia which had a CDR ratio between the richest and poorest of 4.9 in 1992).[19] There was no adjustment for rurality, and no more recent similar data was found for the region.

According to the District Health Barometer[11, 20] reports, the CDR in South African district-level hospitals was 16.15% in 2008/9 and 24.1% in 2015/16. Unfortunately, the reports do not address CDRs in rural settings.

Obstetric/perinatal outcomes in a rural low-resource setting

Very recent evidence involving more than 3500 patients highlights that CDs conducted in the African continent are associated with a 50-fold risk of maternal mortality than those conducted in high-income countries.[21]

There is general consensus that the ideal CDR to achieve optimal outcomes, depends on the setting in which the obstetric care is being delivered, as most high-income health systems are performing excessive CDs without a corresponding decrease in mortality, whilst health systems in low and middle income countries (LMICs) would benefit from increasing their capacity to offer CD (and thus increase CDR) in order to decrease mortality.[22] The exact target is not agreed upon:

- The global data from Molina et al.[23] suggest that the optimal CDR in relation to maternal and neonatal mortality is approximately 19 CDs per 100 live births, which is significantly higher than the WHO recommendation of 10-15 CDs per 100 live births.[24]
- A 2015 systematic review of the global evidence of the association between CD use and

mortality concluded that CD use improved maternal, neonatal, and infant survival until a threshold ranging from 9% to 16%[25]

- Analysis of recent global population-level data, concludes that exceeding a CDR of 10% may not be necessary in order to achieve the lowest maternal and neonatal mortality[26]

When relating the obstetric practices to the outcomes in a LMIC setting, Harrison et al.[27], found that in the African LMIC sites, CD (when compared with vaginal delivery) is associated with an increase in all maternal and perinatal adverse outcomes, whereas in non-African LMIC sites, it is associated with less postpartum haemorrhage and stillbirths. This data was statistically significant and derived from prospective monitoring of almost 400000 deliveries. The authors postulated that either the patients in the African cohort may have been in a worse clinical state on account of the obstetric conditions warranting CD, or that poor quality of CD services was responsible for the observed trends.

Looking more specifically at rural South African obstetric services (albeit at regional level):

- Van Bogaert & Misra[28] found that in a rural regional-level South African hospital, CD did not significantly improve the 5-minute Apgar score when the amniotic fluid is meconium-stained, calling for an improvement in diagnosis of non-reassuring foetal condition, related to meconium-staining.

- Also reporting on data from a rural regional-level South African hospital, and not specifically looking at impact of the mode of delivery on the outcomes, Makhanya et al.[29] identified “foetal distress” as the leading indication for CD, without a corresponding high number of admissions to the neonatal unit for birth asphyxia; this, he postulated, may have been due to either timeous or unwarranted intervention.

Specific to a rural South African setting, two descriptive studies were identified:

- Moalusi[30] examined the clinical outcomes and practices during 2009 in the maternity unit of a rural district-level hospital in North-West Province – within 699 deliveries he found a CDR of 16.3%, an assisted delivery rate of 3.6%, a perinatal mortality rate of 56 per 1000 live births, and a statistically significant relationship between mode of delivery and perinatal outcome (all of the fresh stillbirths and 90% of macerated stillbirths were born by normal vertex delivery).
- Gaunt[31] audited vacuum deliveries occurring in 2014 in a deep rural district-level in the Eastern Cape – within 319 deliveries, he found a CDR of 17.8%, and an assisted delivery rate of 7.4%. The neonatal mortality rate for vacuum deliveries (excluding known stillbirths diagnosed before delivery) was 11.9 per 1000 (higher than the overall institutional neonatal mortality rate of 9.3 per 1000), but no statistical analysis was performed. There were few

recorded maternal complications (mostly perineal tears), but the latter finding being questionable due to the possibility of reporting bias.

Finally, the 7th Saving Mothers (2014-2016)[7] and 10th Saving Babies (2014-2016)[8] reports list some issues specific to rural and/or primary-level facilities:

- The lack of specialist support within the facility, and within the district, to assist non-specialists with decision-making around high-risk cases
- A lower CDR in rural vs. urban provinces
- A higher CFR related to CD in rural vs. urban provinces
- A higher institutional maternal mortality ratio (iMMR) for CD compared to vaginal delivery at primary-level facilities
- A higher iMMR related to anaesthesia in rural vs. urban provinces
- A failure in the referral system, namely not referring to the next level of care timeously, and delays in inter-facility emergency transport
- A larger proportion of avoidable factors related to healthcare workers at district-level facilities compared to higher levels of care

However, there are few recommendations made specifically for rural primary-level facilities to overcome the above. It has been noted that targeted interventions for rural healthcare in South Africa have been few, and that issues which particularly effect rural health facilities, such as a lack of human resources for health, drive avoidable and modifiable factors in maternal and child mortality[32].

Conclusion

Complete consensus does not exist within the literature around target CDR, and this suggests that that a ‘one-size-fits all’ solution is not realistic. It seems rather that the correlation between obstetric/perinatal outcomes and obstetric practice is context specific. There is a paucity of data to further characterise this correlation within the South African rural district-level context. Through addressing this gap in knowledge, a desired outcome of this study is to inform future policies and health system interventions, with a “rural-proof” focus. As such, it has the potential to make a powerful difference in a priority area (Maternal, Neonatal & Child Health), as declared by the National Department of Health, for a key population, namely under-served rural communities.

RESEARCH DESIGN AND METHODS

Study setting

Bethesda Hospital is a district-level hospital, which is located in the rural district of uMkhanyakude, which is in the poorest quintile of South African districts.[33] The hospital is ranked as the 14th most rural out of 255 district-level hospitals in the country.[15] The hospital labour ward and operating theatre conduct approximately 150 deliveries monthly. Twenty-eight percent of deliveries is by CD, but the CDR including deliveries conducted within the clinics draining to the hospital is 22%. [34]

Study design

A cross-sectional observational analytical study.

Target population

Pregnant women using Bethesda Hospital as their site of intrapartum care and delivery.

Study population

All pregnant women who delivered at Bethesda hospital in 2018, according to the labour ward register.

Exclusion criteria

Deliveries which were not conducted within Bethesda Hospital Labour Ward/Operating Theatre.
Neonates with a birth weight of less than 1000g.
Stillbirths diagnosed before the onset of labour.
Files that could not be retrieved.

Sampling

Method of selecting sample: Systematic random probability sampling
Size of sample: To compare the maternal and perinatal outcomes at Bethesda Hospital in 2018 against the modes of delivery, assuming 95% confidence and an acceptable margin of error of 5%, and maximum variability i.e. 50% (given unknown previous comparison), a sample size of 300 patients for each arm was required. The sample was further increased by a margin of 15% to account for potential incomplete documentation or lost files, and multiplied by a design effect (D) of 0.5. Hence, the final sample size of the study was 400 in each arm. Increasing the sample size reduced the type I and type II errors as well as known and unknown cofounders effects. Hence, power of the sample (1-β) and (the % chance of detecting difference) of the study was set at 80%.

Data Sources

Data Sources – Maternity Case Record, labour ward delivery registers, operating theatre record, Bethesda Hospital Caesarean Section audit tool (see Appendix 5), and minutes from monthly perinatal morbidity/mortality meetings

The Caesarean Section audit tool was completed at the doctors' meeting on the morning of the working day following the day of delivery, by the senior doctor allocated to maternity, who made subjective assessments on the urgency and validity of the indication for CD, based on the information presented during the meeting. If (s)he felt unable to come to a conclusion about these variables, the case would be further discussed with another senior doctor, and consensus achieved.

Data Collection Techniques – a retrospective review of above sources, using data collection tool (see Appendix 1); approximately 150 files reviewed per day.

Measures to ensure Validity

Internal validity:

- Information bias due to incomplete documentation will be minimised by cross-referencing information in the Maternity Case Record with delivery and operation registers; data will be collected using a standardised data collection tool, to minimise subjectivity during data collection.
- Information bias due to the data capturer being aware of both the patient's exposure (obstetric practice) and outcome will not be addressed.
- The subjective assessments made during the auditing of CDs is a potential source of information bias. However, this is limited, as the assessor was largely the same person throughout 2018, and reliability in ambiguous cases was improved by consulting with another senior clinician.
- It is noted that there will be several subjective retrospective assessments made in relation to CD (whether it was indicated, how urgent the CD was, and what the optimal mode of anaesthesia was for CD), which is a potential source of information bias. However, this subjectivity will be limited by having one assessor (the principal investigator)
- Additional information bias that is acknowledged but will not be possible to address is related to the quality of documentation in the Maternity Case Record. This is of specific concern when considering under-reporting of adverse events (e.g. low Apgar score neonate or quantity of post-partum haemorrhage); this is also relevant in mothers whose adverse outcomes have required transfer to regional level, in which case the Maternity Case Record is transferred with them, but not returned (photocopying of the file pre-transfer is not always done), and may also be of relevance with Maternity Case Records that have gone missing.

- The impact of confounders to associations, namely variable staffing levels and skill over the course of 2018 will be attempted to be minimised through random sampling, but given the small target population, it will be difficult to eliminate it.
- A source of selection bias is the exclusion of all deliveries which occur in the primary healthcare clinics draining into Bethesda Hospital, as from a clinical governance perspective, deliveries occurring in these clinics are done under the supervision of the hospital. The decision to exclude these deliveries was based on an assessment of feasibility of data collection, given the timeframes and budget for the study.
- Lastly, if an adverse perinatal outcome occurred in the antenatal phase of pregnancy, analysis of these deliveries would lead to selection bias when analysing intrapartum events with outcomes. Hence, exclusion of stillbirths diagnosed before labour is justified.

External validity: the findings of the study will only be generalisable to sites of intrapartum care that have similar features to Bethesda Hospital, as defined in study setting

Pilot study

A pilot study surveying 10% of the final intended sample size, will be carried out at Wentworth Hospital (where the investigator is currently based), to validate the data collection tool. This will only be done once ethical approval has been obtained.

List of Variables to be Measured

Some variables were chosen based on clinical experience and consultation with colleagues, and others were derived from Sauvegraine et al.[35] and Pyykönen et al.[36].

Clinical features:

- Maternal age, weight, HIV status (most recent CD4/viral load if positive), parity and gestational age, other clinical features relating to Robson group, with assignment of Robson group (see Appendix 4)

Obstetric practices:

- Mode of delivery
- For CD: Indication for CD, timing of decision/handover to theatre/anaesthesia start time/operation start time/ operation end time, compliance with WHO checklist, mode of anaesthesia
- Day of delivery (weekday vs. weekend/public holiday), time of delivery

Perinatal outcome:

- Neonatal birthweight and outcome (stillbirth vs. livebirth and Apgar scores at 1 and 5 minutes if livebirth +/- cord blood gas pH if performed)
- Neonatal birth trauma (Erb's palsy, clavicular fracture)

- Neonate admitted to nursery (admission and discharge diagnoses, need for transfer/ventilation, admission for longer than 7 days)

Obstetric outcome:

- Maternal post-partum blood loss volume +/- need for blood transfusion, non-pneumatic anti-shock garment, or laparotomy
- Maternal uterine rupture in women with previous CD
- Duration of post-partum hospital stay
- For vaginal deliveries: third/fourth degree perineal laceration, episiotomy, use of vacuum/forceps
- For CD: wound infection

Freehand record of other significant event in intra-partum/post-partum phase

Assessment of obstetric practice

- Retrospective assessment of whether delivery was conducted at appropriate level of care, in light of provincial referral criteria (see Appendix 3)
- For CD: Lucas class (see Appendix 4), retrospective assessments of whether CD was indicated, whether mode of anaesthesia was the optimal one for the patient according to the clinical profile

Plan for Data Collection

Extract information regarding above variables from Maternity Case Records, cross-referencing the information with delivery and operation registers, and capture this in an epiinfo™ form.

Plan for Data Processing/Handling

Convert data collected via epiinfo™ form into decimal numerals in a Microsoft Excel™ spreadsheet to facilitate statistical analysis. Password-protect data and delete after five years.

Statistical methods

Descriptive statistics will be presented as percentages, frequencies. Associations will be analysed using correlation, chi-squared and T-tests.

List of associations to be Measured

Relationship between the mode of delivery and outcome indicators outlined above.

Relationship between other appropriateness of care (level of care, optimal mode of delivery, optimal mode of anaesthesia), and outcome indicators outlined above.

Subgroup analysis of each Robson group (e.g. Caesarean delivery rate).

ETHICAL CONSIDERATIONS

Institutional ethical review

Approval will be sought from the Biomedical Research Ethics Council of the University of KwaZulu-Natal.

Permissions

Thereafter, gatekeeper permissions will be obtained from the KwaZulu-Natal Health Research & Knowledge Management Directorate via the National Health Research Database, and from the Bethesda Hospital Ethics Committee via the office of the Medical Manager (see Appendix 6).

WORK PLAN

- Time Lines: See Gantt chart in Appendix B
- Budget
  - Stationery: R2000
  - Data collection (fuel, accommodation, data capturer): R15000
  - Conference fees: R8000
  - Publication fees: R10000
  - TOTAL: R35000
- Proposed funding sources:
  - Discovery Foundation R25000
  - UKZN College of Health Sciences bursary R10000

ACKNOWLEDGEMENTS

My supervisors, Dr TC Nkabinde and Prof M Naidoo for their constant guidance and feedback.

The academic Department of Family Medicine in UKZN (including fellow registrars) for their valuable input.

My wife, Jacinth, children, Gabriel and Luca, parents, Mohammed and Małgorzata, and family-in-law, Roy, Jenny, Roxanne and Debashan, for their patience and support.

REFERENCES

1. Shah A, Fawole B, M'Imunya J M, Amokrane F, Nafiou I, Wolomby JJ, et al. Cesarean delivery outcomes from the WHO global survey on maternal and perinatal health in Africa. Int J Gynaecol Obstet. 2009;107(3):191-7.
2. Mathai M, Engelbrecht S. Managing complications in pregnancy and childbirth: a guide for midwives and doctors. 2nd ed. Geneva: World Health Organization; 2017.
3. Maaloe N, Bygbjerg IC, Onesmo R, Secher NJ, Sorensen BL. Disclosing doubtful indications for emergency cesarean sections in rural hospitals in Tanzania: a retrospective criterion-based audit. Acta Obstet Gynecol Scand. 2012;91(9):1069-76.
4. Puchalski Ritchie LM, Khan S, Moore JE, Timmings C, van Lettow M, Vogel JP, et al. Low- and middle-income countries face many common barriers to implementation of maternal health evidence products. J Clin Epidemiol. 2016;76:229-37.
5. Boerma T, Ronsmans C, Melesse DY, Barros AJD, Barros FC, Juan L, et al. Global epidemiology of use of and disparities in caesarean sections. The Lancet. 2018;392(10155):1341-8.
6. Setting minimum standards for safe caesarean delivery in South Africa : review. Obstetrics and Gynaecology Forum. 2015;25(3):41-4.
7. Saving Mothers 2014-2016: Seventh Triennial Report on Confidential Enquiries into Maternal Deaths in South Africa. Pretoria: National Department of Health; 2018.
8. Saving Babies 2014-2016: Triennial report on perinatal mortality in South Africa. Pretoria: National Department of Health; 2018.
9. Betran AP, Ye J, Moller AB, Zhang J, Gulmezoglu AM, Torloni MR. The Increasing Trend in Caesarean Section Rates: Global, Regional and National Estimates: 1990-2014. PloS one. 2016;11(2):e0148343.
10. Massyn N, Day C, Dombo M, Barron P, English R, Padarath A. District health barometer 2012/13. Durban: Health Systems Trust. 2013:1-420.
11. Massyn N, Peer N, English R, Padarath A, Barron P, Day C, editors. District health barometer 2015/16. Durban: Health systems trust. 2016.
12. Burger R, Christian C. Access to health care in post-apartheid South Africa: availability, affordability, acceptability. Health economics, policy, and law. 2018:1-13.
13. Health Do. Human Resources for Health South Africa 2030. Department of Health Pretoria; 2011.
14. Kong VY, Van de Linde S, Aldous C, Handley JJ, Clarke DL. Quantifying the disparity in outcome between urban and rural patients with acute appendicitis in South Africa. South African Medical Journal. 2013;103(10):742-5.
15. Defining Rural – What do we mean by rurality? How rural is your facility? Johannesburg: Rural Health Advocacy Project; 2018 [Available from: http://rhap.org.za/our-projects/defining-rural/.
16. Allanson ER, Pattinson RC. Quality-of-care audits and perinatal mortality in South Africa. Bull World Health Organ. 2015;93(6):424-8.
17. Boatin AA, Schlotheuber A, Betran AP, Moller AB, Barros AJD, Boerma T, et al. Within country inequalities in caesarean section rates: observational study of 72 low and middle income countries. BMJ. 2018;360:k55.
18. Ronsmans C, Etard JF, Walraven G, Hoj L, Dumont A, de Bernis L, et al. Maternal mortality and access to obstetric services in West Africa. Tropical medicine & international health : TM & IH. 2003;8(10):940-8.
19. Ronsmans C, Holtz S, Stanton C. Socioeconomic differentials in caesarean rates in developing countries: a retrospective analysis. Lancet (London, England). 2006;368(9546):1516-23.
20. Day C, Monticelli F, Barron P, Haynes R, Smith J, E S. District Health Barometer 2008/09. Durban: Health Systems Trust. 2010.
21. Bishop D, Dyer RA, Maswime S, Rodseth RN, van Dyk D, Kluyts HL, et al. Maternal and neonatal outcomes after caesarean delivery in the African Surgical Outcomes Study: a 7-day prospective observational cohort study. Lancet Glob Health. 2019;7(4):e513-e22.
22. Betran AP, Temmerman M, Kingdon C, Mohiddin A, Opiyo N, Torloni MR, et al. Interventions to reduce unnecessary caesarean sections in healthy women and babies. Lancet (London, England). 2018;392(10155):1358-68.

23. Molina G, Weiser TG, Lipsitz SR, Esquivel MM, Uribe-Leitz T, Azad T, et al. Relationship between cesarean delivery rate and maternal and neonatal mortality. Jama. 2015;314(21):2263-70.
24. Organization WH. Appropriate technology for birth. Lancet (London, England). 1985;2:436-7.
25. Betran AP, Torloni MR, Zhang J, Ye J, Mikolajczyk R, Deneux-Tharaux C, et al. What is the optimal rate of caesarean section at population level? A systematic review of ecologic studies. Reproductive health. 2015;12:57.
26. Ye J, Zhang J, Mikolajczyk R, Torloni MR, Gulmezoglu AM, Betran AP. Association between rates of caesarean section and maternal and neonatal mortality in the 21st century: a worldwide population-based ecological study with longitudinal data. BJOG : an international journal of obstetrics and gynaecology. 2016;123(5):745-53.
27. Harrison MS, Pasha O, Saleem S, Ali S, Chomba E, Carlo WA, et al. A prospective study of maternal, fetal and neonatal outcomes in the setting of cesarean section in low- and middle-income countries. Acta Obstet Gynecol Scand. 2017;96(4):410-20.
28. van Bogaert LJ, Misra A. Neonatal outcome after caesarean birth for fetal distress and/or meconium staining in a South African rural setting. J Obstet Gynaecol. 2008;28(1):56-9.
29. Makhanya V, Govender L, Moodley J. Utility of the Robson Ten Group Classification System to determine appropriateness of caesarean section at a rural regional hospital in KwaZulu-Natal, South Africa. South African medical journal = Suid-Afrikaanse tydskrif vir geneeskunde. 2015;105(4):292-5.
30. Moalusi O. Clinical outcomes and practices in the maternity unit of a District Hospital 2011.
31. Gaunt CB, editor An Audit of Vacuum Deliveries at Zithulele Hospital. The 34th Conference on Priorities in Perinatal Care in South Africa 2015; Champagne Sports Resort, Drakensberg, KZN2015.
32. Versteeg M, Du Toit L, Couper I. Building consensus on key priorities for rural health care in South Africa using the Delphi technique. Global health action. 2013;6(1):19522.
33. Massyn N, Padarath A, Peer N. District health barometer 2016/17: Health Systems Trust; 2017.
34. Gate K. Perinatal M&M. email ed2018.
35. Sauvegrain P, Chantry AA, Chiesa-Dubruille C, Keita H, Goffinet F, Deneux-Tharaux C. Monitoring quality of obstetric care from hospital discharge databases: A Delphi survey to propose a new set of indicators based on maternal health outcomes. PloS one. 2019;14(2):e0211955.
36. Pyykönen A, Gissler M, Jakobsson M, Petäjä J, Tapper AM. Determining obstetric patient safety indicators: the differences in neonatal outcome measures between different-sized delivery units. BJOG: An International Journal of Obstetrics & Gynaecology. 2014;121(4):430-7.
37. Robson MS. Classification of caesarean sections. Fetal and maternal medicine review. 2001;12(1):23-39.
38. Lucas D, Yentis S, Kinsella S, Holdcroft A, May A, Wee M, et al. Urgency of caesarean section: a new classification. Journal of the Royal Society of Medicine. 2000;93(7):346-50.

APPENDICES

Appendix 1: Data Collection Tool (data will be entered electronically onto an epiinfo™ form)

Unique identifier	Date of delivery + initials +/- number										
Maternal DoB	DDMMYY										
Date of delivery	DDMMYY										
Day of delivery	Weekday/weekend or public holiday										
Time of delivery	MMHH										
Maternal age	Completed years										
Maternal weight	kg										
Maternal HIV status	+/-/unknown	Latest CD4 if +	cells/µl	Latest VL if +	c/ml						
Maternal parity											
Number of foetuses	Singleton/multiple										
Presentation/lie	Cepahlic/breech/other e.g. transverse, oblique										
Previous CS	Yes/No	Uterine rupture if previous CS	Yes/No								
Labour	Spontaneous/induced/ pre-labour CS										
Gestational age	Completed weeks										
Robson group	1-10										
Neonatal birthweight	g										
Birth outcome	Livebirth/stillbirth										
Apgar score at 1 minute	/10										
Apgar score at 5 minutes	/10										
Cord gas pH											
Neonatal birth trauma	Yes(specify)/No										
Neonate admitted to nursery	Yes/No	Admission diagnosis	Freehand	Discharge diagnosis	Freehand	Outcome	Discharged/ died/ transferred out	Mechanical ventilation	Yes/No	Duration of admission	Days
Neonatal HIV status (if mother infected)	Birth PCR negative/positive/not done										
Maternal post-partum blood loss volume	ml	Blood transfusion	Yes/No	NASG	Yes/No	Outcome	Discharged/ died/ transferred out	Mechanical ventilation	Yes/No	Post-delivery laparotomy	Yes/No
Duration of post-partum hospital stay	Days										
Delivery conducted at appropriate level of care	Yes/No										
Vaginal delivery	3rd/4th degree tear	Episiotomy	Vacuum/ forceps								
Caesarean delivery indication	Primary	Secondary									
Caesarean delivery Lucas class	I-IV										
Caesarean delivery indicated at time of decision	Yes/No										
Caesarean delivery timing	Decision time	Handover	Anaesthesia start	Operation start	Delivery	End of operation					
Caesarean delivery WHO checklist compliance	Yes/Partial/No										
Caesarean delivery mode of anaesthesia	Spinal/General/Other (specify)	Optimal mode of anaesthesia	Yes/No Qualify	Anaesthetic complication	Yes (specify)/ No						
Caesarean delivery wound infection	Yes/No										
Any significant events	Freehand										

Appendix 2: Gantt chart

	Jan-19	Feb-19	Mar-19	Apr-19	May-19	Jun-19	Jul-19	Aug-19	Sep-19	Oct-19	Nov-19	Dec-19	Jan-20	Feb-20	Mar-20	Apr-20	May-20	Jun-20	Jul-20	Aug-20	Sep-20	Oct-20	Nov-20	Dec-20	Jan-21	Feb-21	Mar-21	Apr-21	May-21	Jun-21
Conceptualistion of research project	X	X	X																											
Write-up of literature review			X	X	X	X																								
Write-up of methodology					X	X	X	X																						
Meeting with biostatistician						X	X																							
Submission of research protocol to UKZN BREC								X	X																					
Anticipate provisional ethical approval from UKZN BREC										X																				
Solicit letter of support from Bethesda Hospital Ethics Committee										X																				
Submission of research protocol, provisional ethical approval from UKZN BREC, and letter of approval from Bethesda Hospital Ethics Committee to KZN HKRM via NHRD database											X																			
Anticipate ethical approval from KZN HKRM											X																			
Submission of letter of approval from KZN HKRM to UKZN BREC											X																			
Anticipate full ethical approval from UKZN BREC												X																		
Conduct pilot study to validate data collection tool													X	X																
Analyse pilot study results and adjust data collection tool														X	X															
Submit revised protocol (if appropriate) to above Ethical Committees															X															
Apply for funding from Discovery Foundation												X																		
Data collection																X	X													
Data analysis																		X	X	X	X									
Apply for funding from UKZN CHS																									X					
Manuscript writing																						X	X	X	X	X	X	X	X	
Submit final report to Ethical Committees above																									X					
Submit abstract to present findings at conference																										X				
Present findings at conference																											X			
Submit manuscript for publication in journal																													X	

Appendix 3: Provincial referral criteria (page 1)

health

Department:
Health
PROVINCE OF KWAZULU-NATAL

OBSTETRICS AND GYNAECOLOGY
Head Office

Criteria for Referral of Obstetric Patients from District Hospital to Regional Referral Hospital

Emergency Referrals (to labour ward)

- Eclampsia
- Severe pre-eclampsia: i.e. pre-eclampsia with one or more of:
  3+ proteinuria
  BP persistently ≥170 systolic or 110 diastolic
  Signs of imminent eclampsia
  HELLP syndrome
  Renal failure
  Clinical coagulopathy
  Pulmonary oedema
- Abruptio placentae Grade 3 (tense, tender uterus, absent fetal heart beat)
- Preterm labour between 26 and 32 weeks' gestation or estimated fetal weight ≤ 1.5kg (do not transfer if delivery imminent)
- Preterm pre-labour rupture of membranes between 26 and 32 weeks' gestation (estimated fetal weight ≤ 1.5kg)
- Obstructed labour (prolonged labour with intra-uterine sepsis or intra-uterine death)
- Suspected rupture of the uterus (only transfer if the patient can be temporarily stabilized with resuscitation. If the patient cannot be stabilized i.e. remains shocked despite resuscitation, take her to theatre without further delay)
- Placenta praevia with active bleeding (as above, only transfer if the patient can be temporarily stabilized with resuscitation.)
- Pulmonary Oedema
- Jaundice
- Acute third or fourth degree perineal tears (unless there is someone at the District Hospital who has the competence to do the repair on-site)

This list is not exhaustive; emergency referrals for other indications can be discussed telephonically when the need arises.
Please discuss all emergency referrals telephonically with a medical officer, registrar or consultant at the regional / tertiary hospital.
If transport delays are anticipated, please consider helicopter transfer of the patient; discuss this with EMRS.

Appendix 3: Provincial referral criteria (page 2)

Referrals to the next ante-natal clinic at the regional hospital (should be referred with appropriate work-up e.g. blood test results)

Note: A woman who presents in labour at the district hospital with any of the risk factors listed below should be referred to the regional hospital as an emergency unless she is so advanced in labour that she is likely to deliver before she reaches the regional hospital. If she does deliver at the district hospital, then the need for transfer could be reviewed post delivery, and discussed with the regional hospital if necessary.

- Moderate pre-eclampsia: i.e. pre-eclampsia with one or more of:
  proteinuria 2+
  BP persistently >150 systolic or 100 diastolic
- Early-onset pre-eclampsia ≤ 32 weeks' gestation
- Any type of hypertension requiring two or more antihypertensive agents for BP control
- Diabetes in pregnancy: poor control on diet (requiring insulin). For pregnant diabetics on diet, 2-hour post-prandial blood sugar should be <7mmol/litre
- Cardiac disease in pregnancy (stable)
- Other complicated medical disorders in pregnancy, e.g. poorly controlled epilepsy/ asthma/ thyroid disease/ SLE
- Complicated multiple pregnancies: no separating membrane (mono-amniotic), discordant growth, higher order multiple pregnancies (triplets and more), single intra-uterine fetal death. If local ultrasound expertise is poor, all multiple pregnancies can be referred for initial assessment
- Polyhydramnios
- Fetal abnormalities detected on ultrasound
- Anti-Rh or other antibodies to red blood cells
- Severe IUGR (estimated fetal weight <1.5kg)
- Placenta praevia (not currently bleeding)
- Anticipated anaesthetic risk (e.g. severe obesity – weight >120kg)
- Thrombocytopaenia: platelet count persistently <100
- Kyphoscoliosis
- HGSIL or worse on PAP smear
- Suspected DVT
- Ovarian / adnexal mass
- Large uterine fibroids (>10cm diameter) or any fibroids in the lower segment (below the presenting part)
- Previous recurrent stillbirths or neonatal deaths (more than 1)
- Previous stillbirth or neonatal death due to abruptio placentae
- Previous baby with major congenital anomaly

Appendix 3: Provincial referral criteria (page 3)

- 3 or more previous consecutive miscarriages
- Suspected molar pregnancy
- Age 37 and above (only before 22 weeks' gestation), for genetic counseling ± testing, and 1st and /or 2nd trimester ultrasound scanning for anomalies. Once congenital anomalies have been excluded, age alone is not a criterion for further antenatal care or delivery at regional / tertiary level.

This list is not exhaustive; referrals for other indications can be discussed telephonically when the need arises. Please discuss all referrals to the antenatal clinic telephonically either with a doctor from the Obstetrics Department or with a midwife from the antenatal clinic.

Following referral to and assessment at the regional / tertiary hospital according to the above criteria, some women may be referred back, if it is felt that they can be safely managed at district hospital level. In such cases clear instructions about further care should be made by the higher level institution.

Conditions which should <u>not</u> routinely be referred

- Ruptured ectopic pregnancy
- Abruptio placentae with live baby
- Complications of AIDS in pregnancy (e.g. cryptococcal meningitis, PCP pneumonia)
- Mild pre-eclampsia (1+ proteinuria)
- Gestational hypertension
- Preterm labour > 32 weeks' gestation or over 1.5kg

There might be special circumstances in which patients with the above problems may need referral to the regional/ tertiary hospital. In this case the special circumstances would need to be discussed with a doctor from the Obstetrics and Gynaecology Department at the regional/ tertiary hospital.

Neil Moran

DR NF MORAN
HEAD, CLINICAL DEPARTMENT:
OBSTETRICS AND GYNAECOLOGY
DEPARTMENT OF HEALTH: KWAZULU-NATAL

02.12.2011

DR S.M. ZUNGU
HEAD OF DEPARTMENT
DEPARTMENT OF HEALTH: KWAZULU-NATAL

Appendix 4: Robson group and Lucas class

Robson group[37]

1	Nulliparous, single cephalic, ≥ 37 week, in spontaneous labour
2a	Nulliparous, single cephalic, ≥ 37 weeks, induced labour
2b	Nulliparous, single cephalic, ≥ 37 weeks, pre-labour CS
3	Multiparous (excluding prev. CS), single cephalic, ≥ 37 weeks, in spontaneous labour
4a	Multiparous (excluding prev. CS), single cephalic, ≥ 37 weeks, induced labour
4b	Multiparous (excluding prev. CS), single cephalic, ≥ 37 weeks, pre-labour CS
5	Previous CS, single cephalic, ≥ 37 weeks
6	All nulliparous breeches
7	All multiparous breeches (including prev. CS)
8	All multiple pregnancies (including prev. CS)
9	All abnormal lies (including prev. CS)
10	All single cephalic, <37 weeks (including prev. CS)

Lucas class[38]

I	Immediate threat to the life of the woman or foetus
II	Maternal or foetal compromise which is not immediately life-threatening
III	No maternal or foetal compromise, but needs early delivery
IV	Delivery timed to suit woman or staff

Appendix 5: Bethesda Hospital Caesarean section audit tool

BETHESDA HOSPITAL CAESAREAN SECTION AUDIT TOOL

Patient information

Name: Age: Delivery date:

IP number: OP number:

Robson group (see key)

- Singleton □ Multiple □
- Cephalic □ Breech □ Other lie (Transverse/oblique) □
- Nulliparous □ Parous (no uterine scar) □ Parous (uterine scar) □
- Spontaneous labour □ Induced labour □ Pre-labour Caesarean section □
- GA ≥37w □ GA <37w (pre-term) □

Robson Group:

Indication

Primary:

Secondary:

Timing

D: ________ **H:** ________ **A:** ________ **O:** ________ **D:** ________ **E:** ________ *

Working hours** □ Out-of-hours □ Decision-to-delivery interval (mins):

Lucas class of urgency of Caesarean section =

Reason(s) for delay (if applicable):

Initial assessment (at handover meeting)

Indicated □ Not indicated □ Further analysis required □

Done at right time □ Done too early □ Done too late □

Final assessment (if required; justify below)

Indicated □ Not indicated □

Notes (if applicable):

Morbidity (to be reviewed at time of discharge of mother and baby)

WHO checklist used □

Perinatal (e.g. injury, resuscitation needed, Apgar <7 @5 minutes, HIE):

- Apgar score (1 minute, 5 minute):
- Birthweight (kg):
- Admitted to nursery? Y □ N □
- Other issues:

Maternal (e.g. PPH - **complete PPH audit tool**, puerperal sepsis, injury to other organs, failed spinal):

- Blood loss (ml):
- Other issues:

***D** = Time of decision; **H** = Time of handover (maternity to OT staff); **A** = time of spinal/GA induction;
O = start of operation; **D** = Delivery of neonate; **E** = End of operation
**decision-to-delivery to have been completed between 0730–1630hrs on a weekday

Appendix 6: Draft letter to Medical Manager of Bethesda Hospital

ERF 3775 Manor Lakes
Manor Estates
44 Old Main Road
Compensation
Ballito
4399

The Chief Executive Officer
Attention: Dr KR Gate, The Medical Manager
Bethesda Hospital
Private Bag X602
Ubombo
3970
[by email: kellygate@gmail.com]

24th July 2019

Dear Dr Gate,

Re: Retrospective chart review

I am a first-year registrar in Family Medicine, studying for an MMed degree at the University of KwaZulu-Natal. As part of this degree, I am hoping to conduct a research project regarding obstetric practices and obstetric/perinatal outcomes at a rural district-level hospital.

I would like to request permission to collect data at Bethesda Hospital by reviewing the following documents from 2018:

- Maternity Case Records
- Delivery register in labour ward
- Operating theatre record in operating theatre
- Caesarean section audit tools
- Minutes from the monthly perinatal morbidity/mortality meetings

All data that I record would be anonymised in terms of patient identifiers.

I attach provisional ethical approval from the Biomedical Research Ethics Council of the University of KwaZulu-Natal.

If you do grant permission for data collection, I would seek further approval from the KwaZulu-Natal Health Research & Knowledge Management Directorate via the National Health Research Database. Ultimately, I would like to share my findings with the hospital by delivering an oral presentation to the hospital (to highlight good practices, and identify areas for improvement in care), and with the wider community of practice, by publishing the findings in a peer-reviewed journal.

Please do not hesitate to ask for further clarity.

Yours sincerely,

Adam Asghar
MP0692778
