# Supplementary material for: An analysis of obstetric practices and outcomes in a deep rural district hospital in South Africa
Source: PLoS One. 2022 Jan 4;17(1):e0262269. doi: 10.1371/journal.pone.0262269 (PMC8726481; doi:10.1371/journal.pone.0262269)
Supplement: Attachment — Submitted filename: Response to reviewers.pdf [file pone.0262269.s004.pdf]

Adam Asghar
Department of Family Medicine
University of KwaZulu-Natal
238 Mazisi Kunene Rd
Glenwood
Durban
4041
Republic of South Africa

24/09/2021

Dear Dr Pillay,

Re: An analysis of obstetric practices and outcomes in a deep rural district hospital in South Africa (PONE-D-21-22819)

Thank you for the careful review of the above manuscript that raises some valuable points.

I have addressed all points raised and referred to the relevant changes according to the line numbers. Please note, these line numbers are only valid in the 'Revised Manuscript with Track Changes' file; once changes were accepted and track changes switched off (in the Revised paper without tracked changes), the line numbers changed. I have removed the original Manuscript from the submission, so that the PDF can be built accordingly.

Journal requirements

- The revised manuscript has been edited according to PLoS One's style requirements (line 541-542; 566-567)

- Participant consent – see below a response from the Chair of the Biomedical Research Ethics Committee of the University of KwaZulu-Natal, which is an internationally accredited Institutional Review Board.

DW Douglas Wassenaar
RE: FW: Waiver of consent
To: Adam Asghar, Cc: Thandaza Cyril Nkabinde, Mergan Naidoo, Shenuka Singh
26 August 2021 at 19:06
Details

Dear Dr Ashgar,
Current national research ethics guidance in South Africa (DoH, 2015), section 3.5.2.3 permits a registered research ethics committee to grant a waiver of informed consent for research on medical records provided that all identifiers are removed and that patients' interests are not adversely affected. Permission from the institutional custodian of the records is also required. These provisions were satisfied and approved for this study.

This should be acceptable to your reviewer.

Yours sincerely,

Prof D R Wassenaar
Chair
Biomedical Research Ethics Committee
Research Office
Westville Campus
University of KwaZulu-Natal
Ph: +27 31 2604769
BREC@ukzn.ac.za
http://research.ukzn.ac.za/Research-Ethics/Biomedical-Research-Ethics.aspx

UNIVERSITY OF KWAZULU-NATAL
INYUVESI YAKWAZULU-NATALI

- Captions for supporting information have been added to the end of the manuscript (lines 651-654)

- The reference list has been updated to include new papers, in light of the peer review process. No cited papers have been retracted (lines 557-559; 617-620; 624-644).

Please continue to the following page for the Reviewer's/editor's comments.

<u>Reviewer's/editor's comments</u>

<table>
<tr><th>Reviewer's/editor's comments</th><th>Authors' response</th><th>Lines</th></tr>
<tr><td>I suggest that the authors explore indications for caesarean deliveries further and compare complication rates.</td><td>This was an oversight on our part not to look at this in the research objectives. A secondary analysis reveals no statistically significant association between CD indication and complications rates.
<table>
<tr><th>CD indication</th><th>Frequency</th><th>Any complication observed*</th><th>Percentage of group</th><th>Odds ratio</th><th>p-value</th></tr>
<tr><td>Hypertensive disease</td><td>10</td><td>7</td><td>70.0</td><td>2.3 (0.5-11.7)</td><td>0.30</td></tr>
<tr><td>Foetal compromise</td><td>49</td><td>30</td><td>61.2</td><td>1.6 (0.6-4.5)</td><td>0.39</td></tr>
<tr><td>Other</td><td>48</td><td>25</td><td>52.1</td><td>1.1 (0.4-3.1)</td><td>0.88</td></tr>
<tr><td>Previous CD, unsuitable for VBAC</td><td>20</td><td>10</td><td>50.0</td><td>Reference</td><td>Reference</td></tr>
<tr><td>CPD</td><td>43</td><td>21</td><td>48.8</td><td>1.0 (0.3-2.8)</td><td>0.93</td></tr>
<tr><td>Post-dates</td><td>14</td><td>5</td><td>35.7</td><td>0.6 (0.1-2.3)</td><td>0.41</td></tr>
<tr><td>Unsuccessful VBAC (prolonged latent phase/poor progress)</td><td>11</td><td>3</td><td>27.3</td><td>0.4 (0.1-1.8)</td><td>0.23</td></tr>
</table>
*Maternal (transfer out, prolonged stay, PPH, puerperal infection, anaesthetic complication); neonatal (admission including transfer out/death, low Apgar, birth trauma)

We have not included the above table in the manuscript. No literature was found to highlight previously described complication rates relating to CD indication.</td><td>265-266
434-437</td></tr>
<tr><td>Authors should compare maternal and neonatal outcomes for each of the Lucas classes.</td><td>Thank you for bringing this point up, which is important. A secondary analysis reveals no statistically significant association between Lucas classes and maternal/complication rates.
<table>
<tr><th>CD Lucas class</th><th>Frequency</th><th>Maternal complications observed*</th><th>Percentage of group</th><th>OR</th><th>p-value</th><th>Neonatal complications observed*</th><th>Percentage of group</th><th>Odds ratio</th><th>p-value</th></tr>
<tr><td>IV</td><td>50</td><td>17</td><td>34.0</td><td>Reference</td><td>Reference</td><td>9</td><td>18.0</td><td>Reference</td><td>Reference</td></tr>
<tr><td>III</td><td>17</td><td>9</td><td>52.9</td><td>2.2 (0.7-6.7)</td><td>0.17</td><td>2</td><td>11.8</td><td>0.6 (0.1-3.1)</td><td>0.27</td></tr>
<tr><td>II</td><td>128</td><td>52</td><td>40.6</td><td>1.4 (0.7-2.7)</td><td>0.36</td><td>32</td><td>25.0</td><td>1.6 (0.7-3.6)</td><td>0.27</td></tr>
<tr><td>I</td><td>0</td><td>N/A</td><td>N/A</td><td>N/A</td><td>N/A</td><td>N/A</td><td>N/A</td><td>N/A</td><td>N/A</td></tr>
</table>
*Maternal (transfer out, prolonged stay, PPH, puerperal infection, anaesthetic complication); neonatal (admission including transfer out/death, low Apgar, birth trauma)

We have not included the above table in the manuscript. Most literature compares emergency vs. elective CD, rather than the individual Lucas classes.</td><td>265-266
434-437</td></tr>
</table>

Avoidable factors such as administrative/or medical related should also be determined in cases where complications occurred. These have been shown in reports from the National Committee on Confidential Enquiries into Maternal Mortality in South Africa to be substantial causes of institutional maternal mortality.	Unfortunately, data was not collected regarding these, as they were not part of the study's objectives. This is, however, a pertinent point. From the data sources available, it would have been difficult to ascertain avoidable factors, as cases of maternal and neonatal morbidity were not discussed at facility Perinatal Morbidity and Mortality Meetings in the same depth as mortality (information bias). We acknowledge that severe acute maternal and neonatal morbidity needs to be thoroughly investigated, and have thus included this oversight as a limitation of the study.	481-483

Other points

The peer review input encouraged us to thoroughly re-examine the whole article, and thus further revisions have been made as follows:

- More accurate description of statistical analysis
  - Lines 18-22, 274, 295, 307, 310, 335-336, 369, 381, 472
  - Table 5, Table 6
- Correction of statistical analyses
  - Lines 131, 143, 203, 325, 347, 363, 419
- Consistency of abbreviations/capitalisation/parentheses
  - Lines 41, 102, 105, 111-112, 225, 309, 368, 372, 439, 441, 447, 485
- Grammatical/spelling correction
  - Lines 76, 159-160, 186, 193, 292, 401, 423, 497
- Points of clarity, including definitions
  - Lines 92, 127, 168-169, 170-171, 177-178, 191, 208, 216-217, 224-225, 252, 254-255, 338, 364-365, 424-433, 502
- Ensuring that all data mentioned in Discussion has been presented in Results
  - Lines 170-174, 190, 236-249, 257-261
  - Table 3
- Additions to Discussion, excluding those addressing Reviewer's/Editor's comments
  - Lines 421-424, 475-476
- No changes were made to the figures originally submitted

Thank you once again for your consideration of this manuscript.

Yours sincerely,

Adam Asghar (on behalf of co-authors Thandaza Nkabinde & Mergan Naidoo)
